# Supplementary material for: pH-Responsive Drug Delivery and Imaging Study of Hybrid Mesoporous Silica Nanoparticles
Source: Molecules. 2022 Oct 2;27(19):6519. doi: 10.3390/molecules27196519 (PMC9572296; doi:10.3390/molecules27196519)
Supplement: Supplementary file 1 [file molecules-27-06519-s001.zip › molecules-1915661-supplementary.pdf]

Supplementary Data

pH-responsiive drug delivery and imaging study of hybird mesoporous silica nanoparticles

Zhongtao Li[1], Jing Guo[2], Guiqiang Qi[1], Meng Zhang[3], Liguo Hao[1 2]*

1. Department of Molecular Imaging, School of Medical Technology, Qiqihar Medical University, Qiqihar, Heilongjiang, 161006, China

2. Department of Molecular Imaging, The First Affiliated Hospital of Qiqihar Medical University, Qiqihar, Heilongjiang, 161041, China

3. Qiqihar Medical University, Qiqihar, Heilongjiang, 161006, China

*Corresponding author (LG H): E-mail: haoliguo@qmu.edu.cn

List of contents:

Page

Figure S1 .... **S3**

Figure S2 .... **S4**

Figure S3 .... **S5**

Figure S4 .... **S6**

Figure S5 .... **S7**

Figure S6 .... **S8**

Figure S7 .... **S9**

Figure S8 .... **S10**

Figure S9 .... **S11**

Figure S10 .... **S12**

Figure S11 .... **S13**

Figure S12 .... **S14**

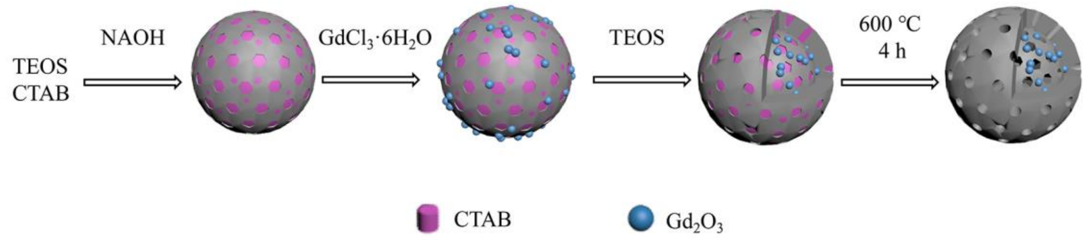

Figure S1. Illustration of the preparation of Gd^{3+}-incorporated mesoporous silica nanoparticles (MSN) (Gd_2O_3@MSN) particles for magnetic resonance imaging

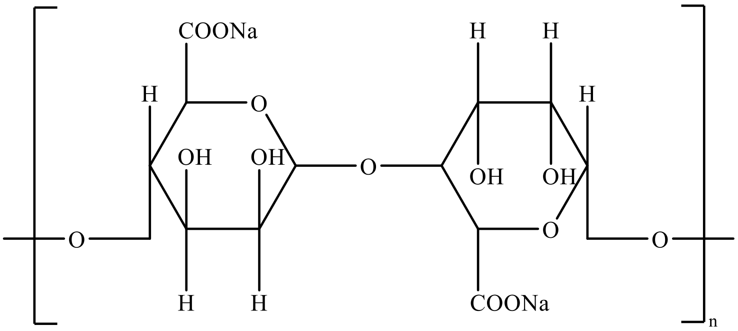

Figure S2. Molecular structure of sodium alginate (SA).

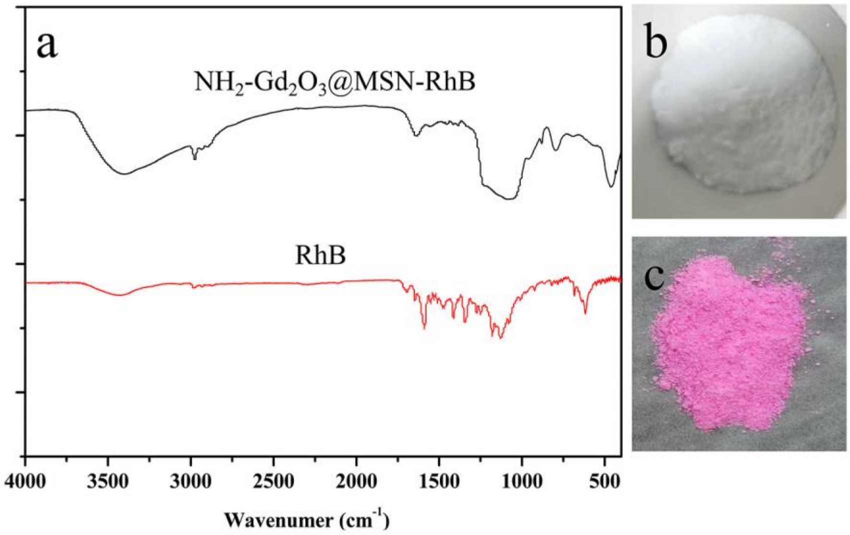

Figure S3. (a) FT-IR spectra of NH_2-Gd_2O_3@MSN-RhB and RhB. Macroscopic images of (b) NH_2-Gd_2O_3@MSN NPs and (c) NH_2-Gd_2O_3@MSN-RhB NPs .

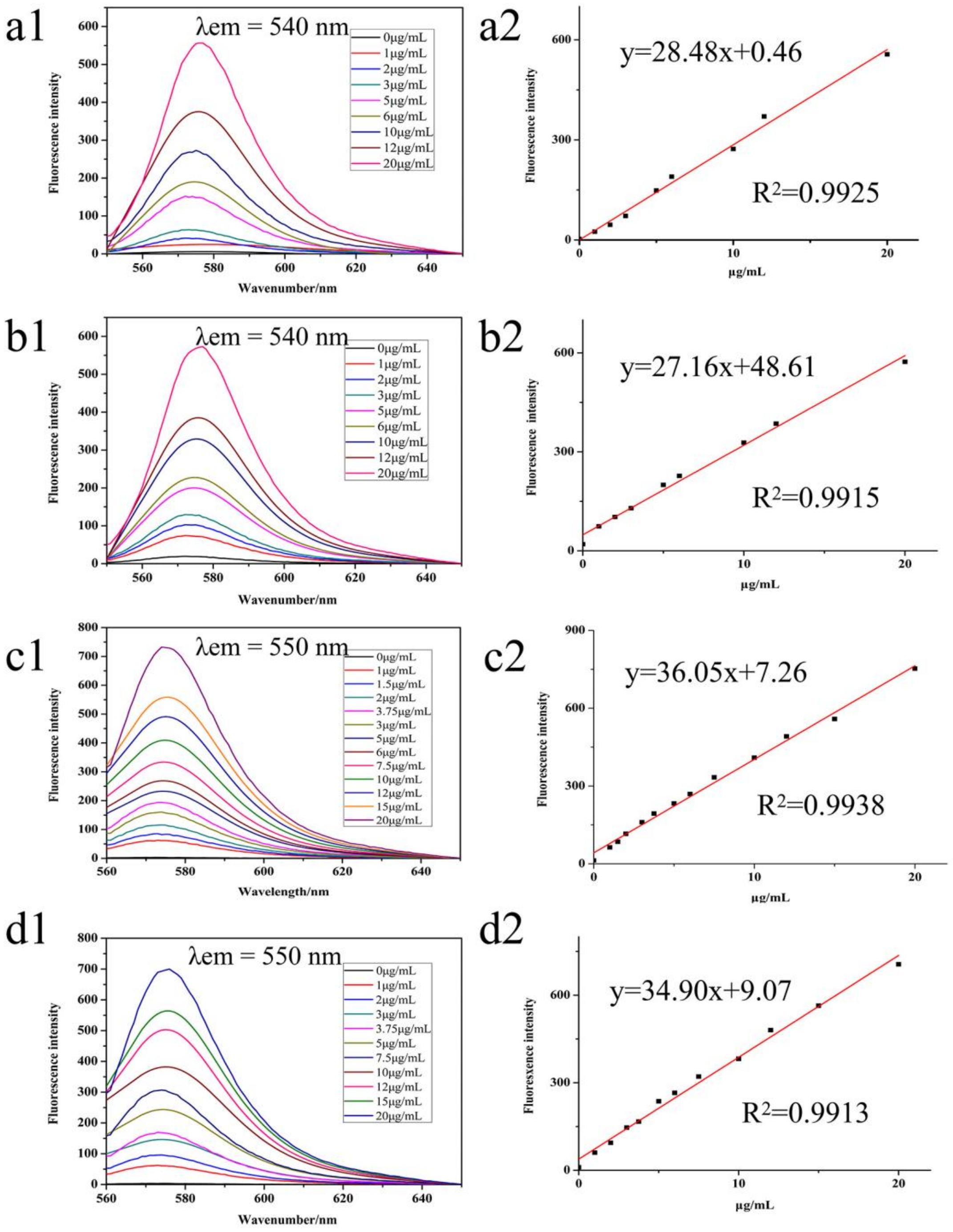

Figure S4. (a1-d1) Fluorescence response of RhB at different solution (ddH_2O, pH 7.4, pH 5.5 and pH 4.5 buffer) and concentrations in the 0.0–20.0 μg/mL rang, respectively. (a2-d2) Calibration curve of RhB.

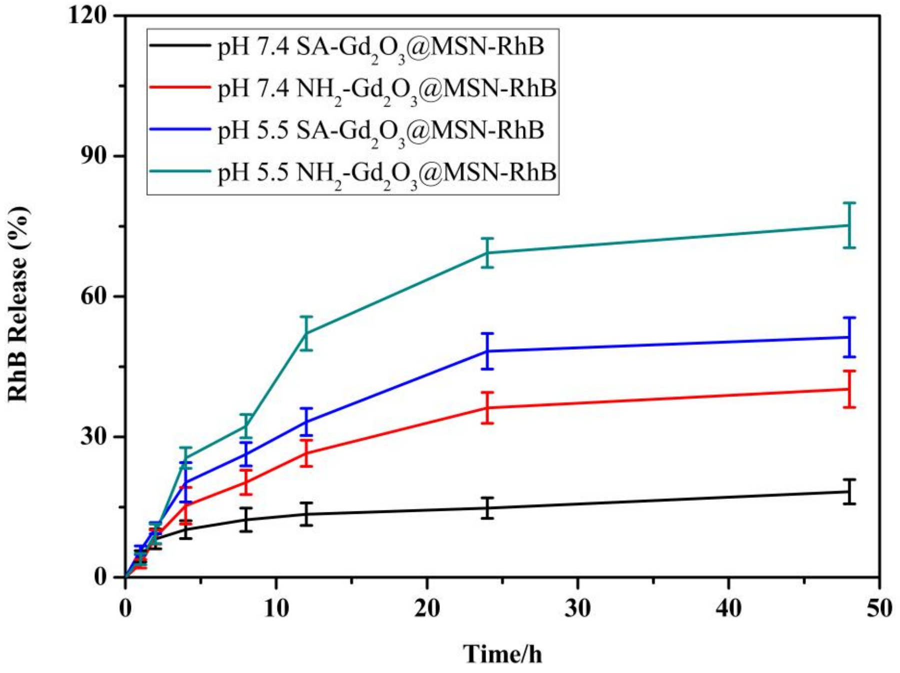

Figure S5. Release curves of SA-Gd_2O_3@MSN-RhB and NH_2-Gd_2O_3@MSN-RhB at different pH values.

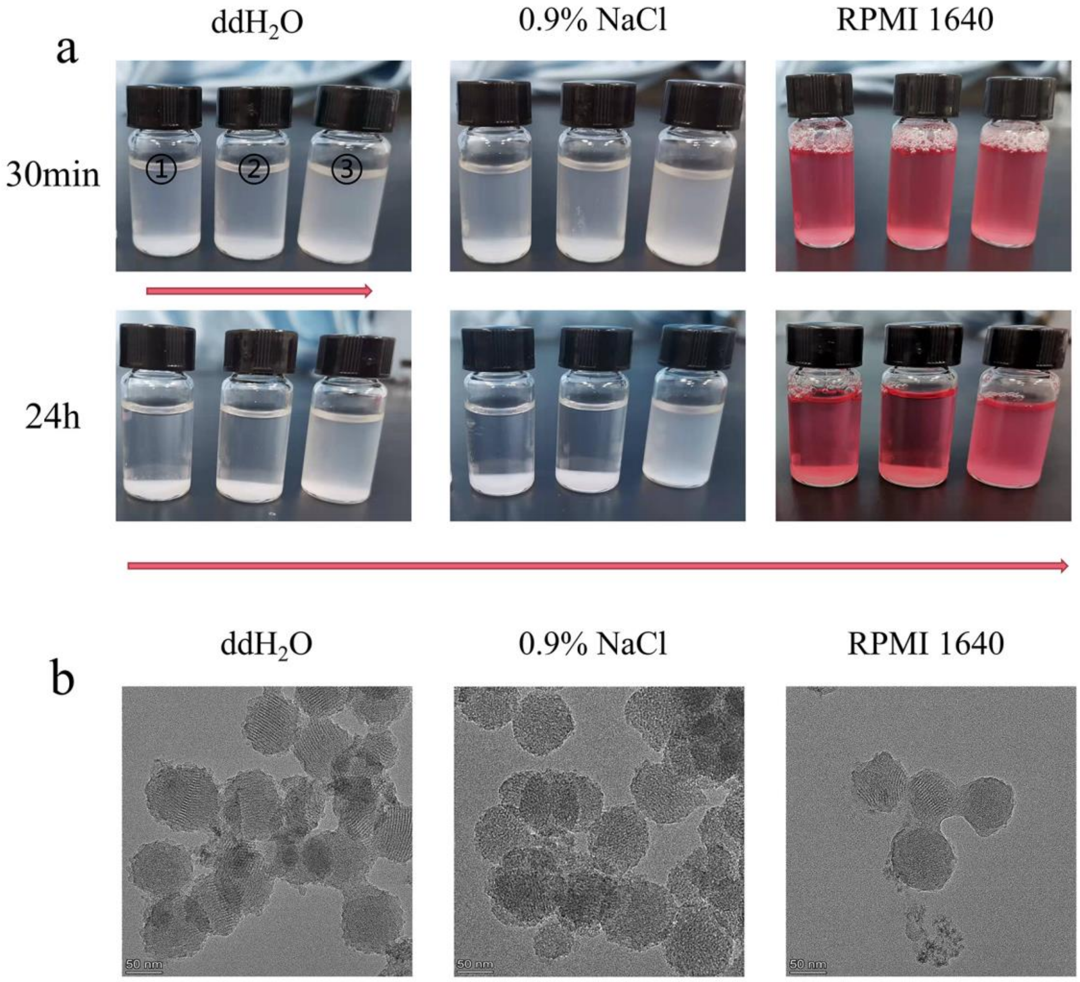

Figure S6. Colloidal stability of SA-Gd_2O_3@MSN. (a) Photograph images of Gd_2O_3@MSN, NH_2-Gd_2O_3@MSN and SA-Gd_2O_3@MSN dispersed in water (①), saline (②) and medium (③) with a concentration of 2 mg/mL. (b) TEM images of SA-Gd_2O_3@MSN after 24 h incubation.

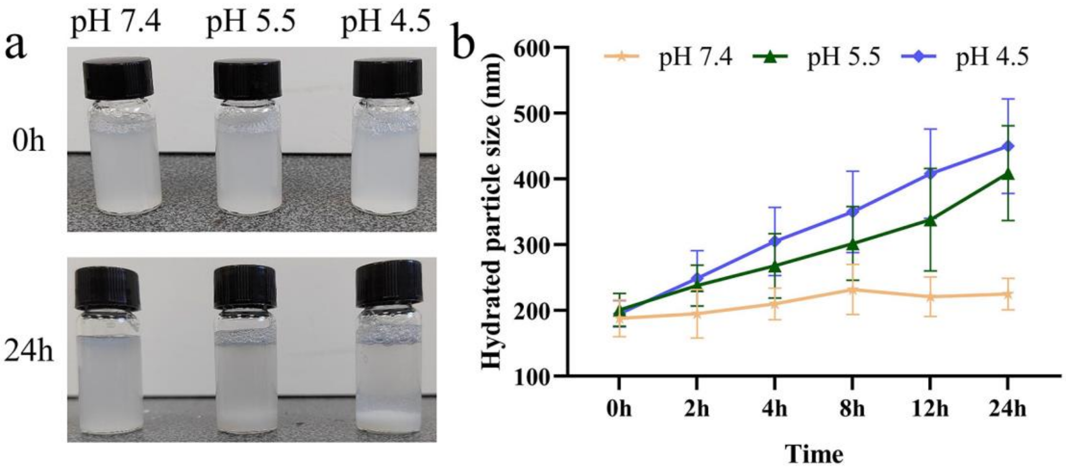

Figure S7. Colloidal stability of SA-Gd_2O_3@MSN at different pH values. (a) SA-Gd_2O_3@MSN in acid solution (pH 5.5, 4.5) begin to aggregate within 2 hour, reaching aggregates hydrodynamic diameters of approximately 400 nm and 450 nm, respectively, after 24 hours of incubation. (b) In contrast, SA-Gd_2O_3@MSN at the ph 7.4 solution do not aggregate over 24 hours. (c) This point is further demonstrated visually after 24 hours when most SA-Gd_2O_3@MSN have sedimented in acid solution and remain transparent in pH 7.4 solution (well-suspended).

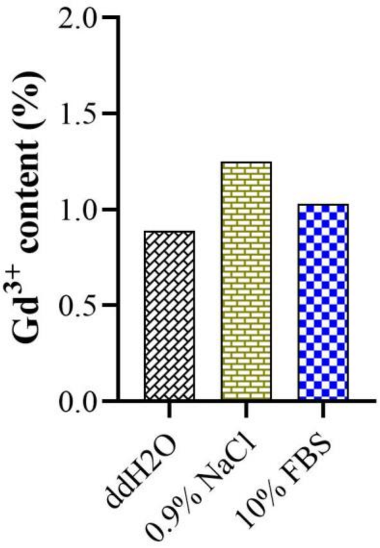

Figure S8. Release of Gd^{3+} in different buffers.

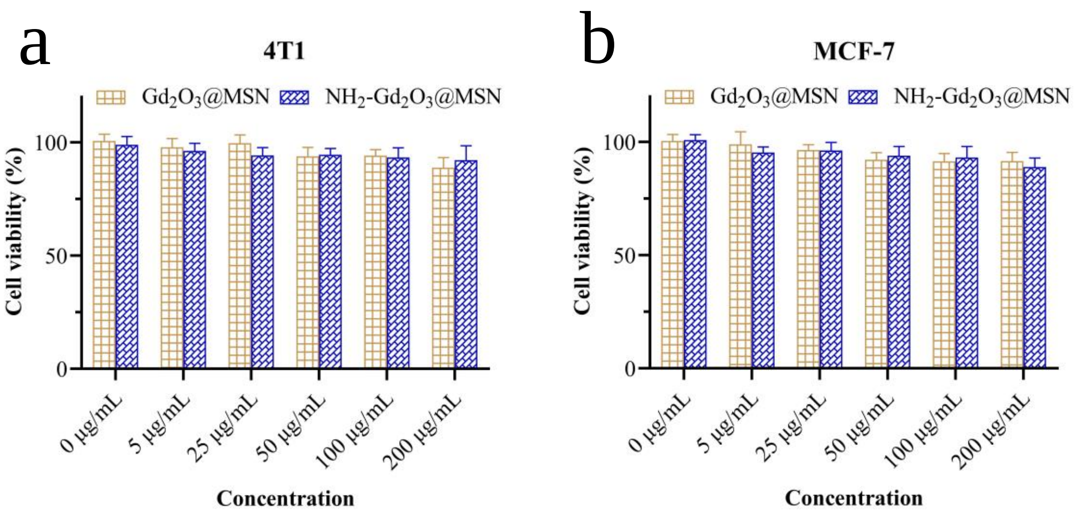

Figure S9. Cytotoxicity against 4T1 (a) and MCF-7 (b) cells after incubation with different concentrations of various NPs for 24 h.

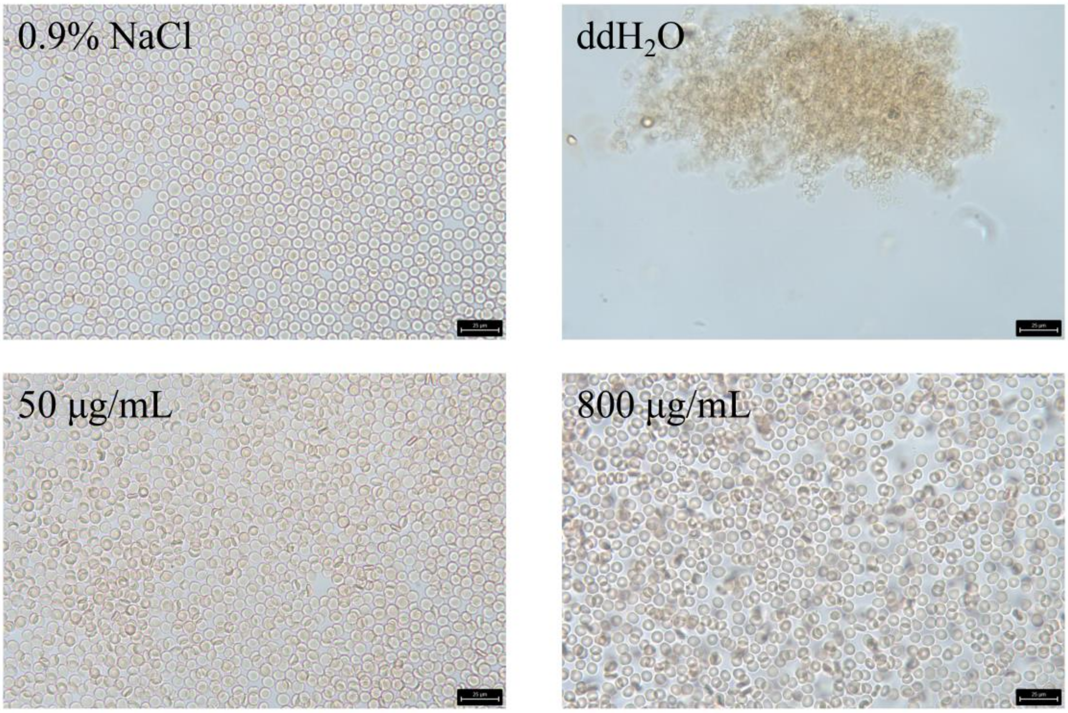

Figure S10. Morphology of human red blood cells upon treatment with 0.9% NaCl, ddH_2O and SA-Gd_2O_3@MSN (50, 800 μg/mL) for 4 h.

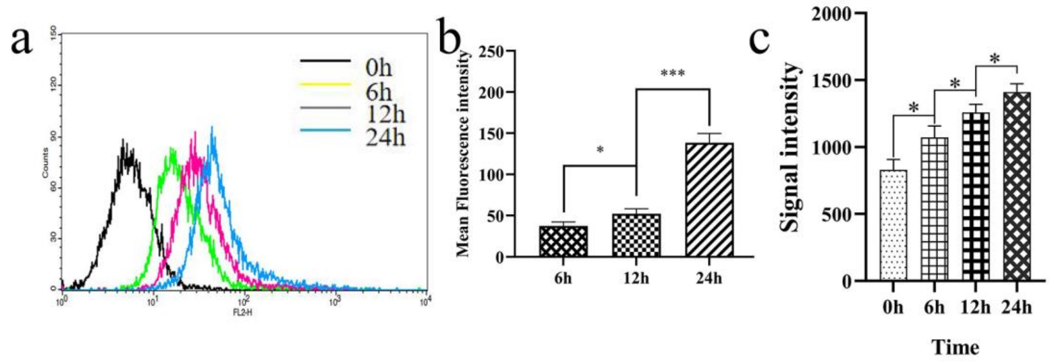

Figure S11. (a) FCM images of 4T1 cells with the treatment of SA-Gd_2O_3@MSN-RhB NPs for 0, 6, 12 and 24 h. (b) Mean fluorescence intensity corresponding to (Fig. 6a). (c) MRI intensity of 4T1 cells after incubation with SA-Gd_2O_3@MSN-RhB at different times. (*$p < 0.05$, ***$p < 0.001$).

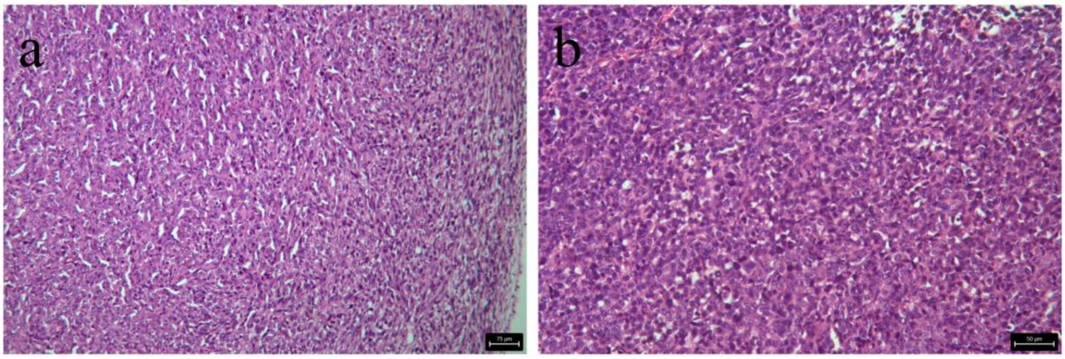

Figure S12. H&E staining of 4T1 xenografts in Balb/c mice.
